# Effect of Human Papillomavirus Vaccine to Interrupt Recurrence of Vulvar and Anal Neoplasia (VIVA): A Trial Protocol

**DOI:** 10.1001/jamanetworkopen.2019.0819

**Published:** 2019-04-12

**Authors:** Helen C. Stankiewicz Karita, Kirsten Hauge, Amalia Magaret, Constance Mao, Jeffrey Schouten, Verena Grieco, Long Fu Xi, Denise A. Galloway, Margaret M. Madeleine, Anna Wald

**Affiliations:** 1 Department of Medicine, University of Washington, Seattle; 2 Department of Biostatistics, University of Washington, Seattle; 3 Division of Vaccine and Infectious Diseases, Fred Hutchinson Cancer Research Center, Seattle, Washington; 4 Department of Laboratory Medicine, University of Washington, Seattle; 5 Department of Obstetrics and Gynecology, University of Washington, Seattle; 6 Department of Surgery, University of Washington, Seattle; 7 Department of Pathology, University of Washington, Seattle; 8 Department of Epidemiology, University of Washington, Seattle; 9 Division of Human Biology, Fred Hutchinson Cancer Research Center, Seattle, Washington; 10 Public Health Sciences, Fred Hutchinson Cancer Research Center, Seattle, Washington

## Abstract

**Importance:**

Human papillomavirus (HPV), particularly HPV type 16, causes most anal and vulvar high-grade squamous intraepithelial lesions (HSIL), which are precursors to cancer. After initial treatment of HSIL, more than 30% of patients will have disease recurrence, with even higher recurrence among HIV-positive individuals and men who have sex with men. Recurrences can be debilitating and lead to significant morbidity and medical expense. Observational studies suggest a possible therapeutic benefit of the licensed HPV vaccines in reducing recurrent lesions in previously infected persons.

**Objective:**

To test whether the licensed prophylactic HPV vaccine (Gardasil-9) can reduce the risk of HSIL recurrence by 50% in previously unvaccinated individuals recently treated for anal or vulvar HSIL.

**Design, Setting, and Participants:**

This is a trial protocol for a randomized, double-blind, placebo-controlled, proof-of-concept clinical trial. Eligible participants are aged 27 to 69 at study start and have not received prior HPV vaccination, have had anal or vulvar HSIL diagnosed on or after January 1, 2014, and have no evidence of HSIL recurrence at screening. Persons infected with HIV are eligible for the study provided they are receiving antiretroviral therapy. Target enrollment is 345 individuals. The primary outcome is time to histopathologically confirmed recurrence of HSIL. Differences in the risk for recurrence of HSIL will be evaluated using Cox proportional hazard models. Additional analyses include (1) frequency of HSIL recurrence; (2) role of HPV antibodies in deterring recurrence; (3) role of HPV persistence in recurrence, as measured by HPV genotype or HPV-16 variant lineage determined using swab samples collected at months 0, 18, and 36; and (4) incidence of adverse events. The study will be conducted at the University of Washington Virology Research Clinic from 2017 through 2022. Participants will be followed up for up to 36 months in the clinic, and up to 42 months by telephone.

**Discussion:**

Management of persistent or rapidly recurring anogenital HSIL remains challenging. Results from this study will provide evidence on whether incorporating the nonavalent HPV vaccine into routine care can decrease recurrence of anal and vulvar HSIL.

**Trial Registration:**

ClinicalTrials.gov identifier: NCT03051516

## Key Points

**Question:**

Can the prophylactic human papillomavirus vaccine (Gardasil-9) reduce the risk of anal and vulvar high-grade squamous intraepithelial lesion (HSIL) recurrence after treatment of the prevalent lesion?

**Findings:**

This trial protocol describes a randomized clinical trial targeting enrollment of 345 HIV-positive and HIV-negative adults with history of anal or vulvar HSIL. The trial seeks to evaluate whether the human papillomavirus vaccine can reduce the risk of recurrence by 50% in previously unvaccinated individuals with a prior histologically confirmed anal or vulvar HSIL diagnosis.

**Meaning:**

If successful, the licensed human papillomavirus vaccine could be incorporated with other treatment for patients with anal and vulvar HSIL to reduce the high rate of HSIL recurrence.

## Teaser

This trial protocol describes a randomized clinical trial to assess the effectiveness of a human papillomavirus vaccine to reduce the risk of anal and vulvar high-grade squamous intraepithelial lesion recurrence after treatment.

## Introduction

Persistent infection with oncogenic human papillomavirus (HPV) has been linked to 70% of vulvar and 90% of anal cancers, causing more than 45 000 cases worldwide each year.^1^ In the United States, more than 10 000 cases are diagnosed annually, and most are HPV-16 related.^2^ Incidence rates of anal and vulvar cancer have increased over the past decades in the United States, particularly among high-risk groups.^3^ Specifically, among US HIV-infected men who have sex with men (MSM), the incidence of anal cancer (78 of 100 000) currently exceeds the incidence of cervical cancer in sub-Saharan Africa (55 of 100 000 women).^4,5^ Locally invasive anal and vulvar cancers are associated with 48% and 59% 5-year survival, respectively.^6^

Persistent HPV infection and high-grade squamous intraepithelial lesions (HSIL) are presumed to lead to HPV-related anal and vulvar cancer, analogous to the natural history of cervical HPV infections leading to cervical cancer.^5,7,8,9^ Incidence rates of anal and vulvar carcinoma in situ, which account for most HSIL in the United States, were 1.0 per 100 000 persons and 3.9 per 100 000 women, respectively, in 2015.^10^ The annual percentage from 2000 to 2015 increased 7.1% for anal HSIL and 0.4% for vulvar HSIL.^10^ Treatment is generally recommended for women with vulvar HSIL.^11,12^ However, no secondary prevention strategies to prevent progression of anal HSIL to invasive cancer have been shown to be effective, although current trials are assessing the potential utility of screening and treatment for anal HSIL (NCT02135419 and NCT02007421). Treatment of anal and vulvar HSIL is variable across centers and includes ablation, excision, or topical therapies with immunomodulating agents. Following treatment for HSIL at either site, approximately 50% of lesions will recur within 5 years following treatment.^13,14,15,16,17,18,19,20,21^ Table 1 shows variation in time to recurrence. In addition, recurrence rates are reported up to 90% among HIV-infected MSM. Recurrent lesions necessitate repeated treatments and may lead to compromised quality of life, functional deficits, and high medical costs.^16^

**Table 1.** Published Studies Assessing Time to Recurrence of Anal and Vulvar HSIL After Treatment

Source	Cohort Size, No.	HIV-Positive Patients, No.	Treatment Modality	Patients With Recurrence After Treatment, %	Time to Recurrence, Median, mo
**Anal HSIL**					
Pineda et al,^17^ 2008	246	182	Surgical	57	19
Goldstone et al,^20^ 2011	96	44	Infrared coagulation ablation	62 (HIV-negative)	14 (HIV-negative)
				91 (HIV-positive)	17 (HIV-positive)
Richel et al,^13^ 2013	388	388	Topical and electrocautery treatment	67	18
**Vulvar HSIL**					
Modesitt et al,^18^ 1998	73	Unknown	Excisional or surgical ablation	46 (If positive surgical margins), 17 (if negative surgical margins)	15 (In positive surgical margins)
					41 (In negative surgical margins)
Jones et al,^7^ 2005	405	5	Excisional or surgical ablation	50 (If positive surgical margins), 15 (if negative surgical margins)	60
Wallbillich et al,^19^ 2012	303	Unknown	Excisional, topical, and surgical ablation	28.7	25
Fehr et al,^15^ 2013	464	Unknown	Excisional, topical, and surgical ablation	30	12
Madeleine et al,^21^ 2016	65	Unknown	Surgical ablation	33.8	36
Satmary et al,^14^ 2018	784	2	Excisional, topical, and surgical ablation	26.3	16.9

Abbreviation: HSIL, high-grade squamous intraepithelial lesions.

The prophylactic bivalent (2vHPV), quadrivalent (4vHPV), and nonavalent (9vHPV) HPV vaccines are safe and effective in reducing HPV-driven cervical cancers by preventing HPV acquisition in HPV-naive persons.^22^ As demonstrated by studies using the 4vHPV vaccine among HIV-infected patients, the prophylactic vaccine (Gardasil-4) is also safe and immunogenic in this group of persons.^23,24^ Although HPV vaccination in persons older than 26 years is not routinely recommended by US guidelines, clinical trials^25,26,27^ demonstrated that the 2vHPV and 4vHPV vaccines are safe, immunogenic, and highly effective in women up to age 55 years. Safety, tolerability, and immunogenicity trials of the 9vHPV vaccine in adult immunocompromised participants are currently ongoing (NCT03036930, NCT03284866, NCT03391921, and NCT03482739).

Several observational studies^28,29,30,31^ suggest that the vaccine may also reduce the risk of HSIL recurrence in previously infected patients. The exact protective mechanism in infected persons is not well understood. One hypothesis is that the vaccine will prevent new infections.^28,30^ The antibody response elicited with the HPV vaccine may prevent cell reinfection and subsequent neoplasia.^32,33^ In addition, the HPV vaccine stimulates cell-mediated immunity,^34,35,36^ which may also play a role in preventing recurrent infection.

## Methods

### Overview of the Trial Design

**Figure 1.**
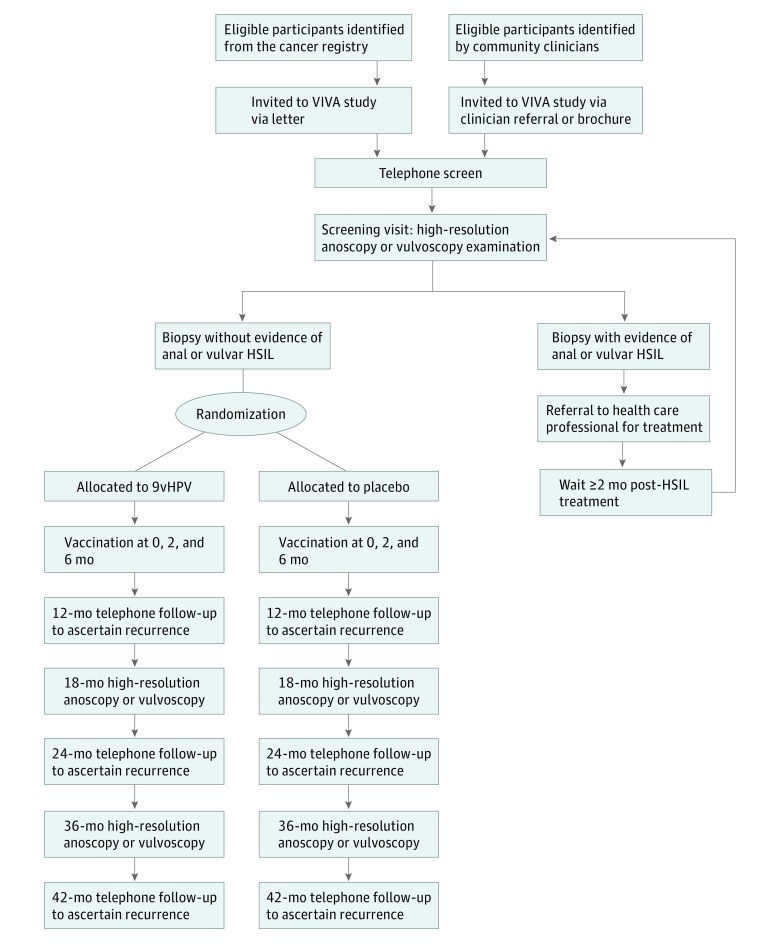
CONSORT Diagram of the Vaccine to Interrupt Progression of Vulvar and Anal Neoplasia (VIVA) Trial HSIL indicates high-grade squamous intraepithelial lesions; 9vHPV, nonavalent human papillomavirus vaccine.

The VIVA (Vaccine to Interrupt Progression of Vulvar and Anal Neoplasia) trial is a randomized, double-blind, placebo-controlled, proof-of-concept clinical trial (Figure 1). The objective is to test whether the 9vHPV vaccine delivered after previously diagnosed anal or vulvar HSIL reduces the hazard of HSIL recurrence by 50% in the vaccinated vs placebo groups over 36 months. Potential participants with a history of anal or vulvar HSIL diagnosed on or after January 1, 2014, may be enrolled if they are HSIL-free at high-resolution anoscopy (HRA) or vulvoscopy examination conducted during the screening visit. Participants are randomized by specific strata (anatomic site of HSIL, HIV status, and time since diagnosis of the qualifying HSIL lesion) in equal proportion to 9vHPV vaccine or placebo, with doses administered during visits at 0, 2, and 6 months. The primary end point is histologically documented recurrence of HSIL. Participants who received the placebo will be offered the 9vHPV vaccine at the end of the study. The study protocol and statistical plan are available in Supplement 1.

### Study Setting and Eligibility Criteria

Participants are evaluated at the University of Washington Virology Research Clinic. This trial was approved by the Fred Hutchinson Cancer Research Center institutional review board and registered at ClinicalTrials.gov (NCT03051516). Eligible participants include men and women who meet the following criteria: (1) age 27 to 69 years; (2) histologically confirmed anal or vulvar HSIL (AIN2/3 or VIN2/3) diagnosed on or after January 1, 2014, with p16^Ink4^ testing if needed to confirm HSIL as determined by the study pathologist; (3) 2 or more months since last therapy for HSIL; (4) no clinical or histological evidence of HSIL on screening examination, with patients whose screening visit reveals HSIL eligible to be rescreened 2 or more months after therapy; (5) residence in the area and willing to attend all visits; (6) for women of childbearing potential, agreement to use effective contraception through study month 7; (7) for HIV-positive persons, treatment with antiretroviral therapy for at least 6 months prior to enrollment; and (8) participant written informed consent and agreement to sign medical records release forms. Criteria for exclusion are the following: (1) pregnancy; (2) chemotherapy (current, within the last month, or anticipated within the first 7 months of enrollment); (3) history of invasive HPV-related cancer; (4) unstable medical conditions (eg, malignant hypertension or poorly controlled diabetes); (5) prior HPV vaccination; (6) allergy or intolerance to lidocaine; (7) current participation in an HPV-related interventional research study (except the Topical or Ablative Treatment in Preventing Anal Cancer in Patients With HIV and Anal High-Grade Squamous Intraepithelial Lesions [ANCHOR] study [NCT02135419], which allows coenrollment with VIVA); and (8) any other condition that, in the opinion of the investigators, may compromise the participant's ability to follow study procedures and safely complete the study (eg, difficult peripheral venous access). Note that patients with immunosuppressive conditions, eg, solid-organ transplant, are not excluded if they otherwise meet the study inclusion criteria.

### Recruitment

Potential participants are primarily identified through the Cancer Surveillance System of the Fred Hutchinson Cancer Research Center, a population-based National Cancer Institute Surveillance, Epidemiology, and End Results cancer registry. The Cancer Surveillance System provides abstracted information on the qualifying lesion site, including lesion behavior, histology, and participant demographics for persons with HSIL. The Surveillance, Epidemiology, and End Results registry identifies approximately 230 patients with AIN3 or VIN3 per year in the catchment region of the study. Some participants are referred to the trial by area physicians.

Participants identified through the Cancer Surveillance System are mailed a letter inviting them to join the study or opt out of further contact. After 10 days, the potential participant is contacted via telephone to arrange a screening appointment. Participants identified through a physician referral are contacted via telephone to set up a screening visit. Partial waivers of Health Insurance Portability and Accountability Act authorization and consent were granted to allow review of electronic medical records to obtain contact information.

### Intervention and Study Visits 

#### Screening Visit

After written informed consent is obtained, participants provide demographic information and medical history. Participants with unknown HIV status are tested for HIV and women of childbearing potential undergo a pregnancy test. A physical examination is performed and anal or vulvar swabs for HPV DNA detection are collected. An HRA or vulvoscopy is performed, depending on the location of the qualifying lesion. Women with vulvar HSIL as their qualifying lesion are offered HRA. A biopsy is obtained if any lesion is visualized by HRA or vulvoscopy examination at the qualifying site. Only those potential participants with a negative biopsy for HSIL or with no clinical evidence of HSIL by HRA or vulvoscopy are eligible for enrollment. Participants with positive biopsy are referred to their health care clinician for treatment; the study does not provide treatment for HSIL. Persons who screen positive may be rescreened once, at 2 months or more following treatment (Table 2).

**Table 2.** Vaccine to Interrupt Progression of Vulvar and Anal Neoplasia Trial Procedures

Procedure	Study mo									
	Screen	Enrollment (mo 0)	2	6	7	12	18	24	36	42
Obtain informed consent	X									
Review inclusion and exclusion criteria	X	X								
Medical history and physical examination	X									
Interim history and physical examination		X	X	X	X		X		X	
HIV testing (as needed)	X									
Urine pregnancy test	X	X	X	X						
Randomization		X								
Vaccination		X	X	X						
Self-administered questionnaire		X		X			X		X	
Blood draw for human papillomavirus antibody		X			X		X		X	
Blood draw for DNA (optional)					X					
High-resolution anoscopy or vulvoscopy	X						X		X	
Anal or vulvar swab for human papillomavirus	X						X		X	
Biopsy	X^a^						X^a^		X^b^	
Telephone visit to ascertain recurrence						X		X		X

^a^ Biopsy, as needed, if lesion is noted during high-resolution anoscopy or vulvoscopy.

^b^ A biopsy will be performed at site of any visible lesion or at the site of qualifying lesion if no lesion is visible.

#### Enrollment Visit and Vaccine Administration

During the clinic visits, a medical history and self-administered questionnaire are collected and a physical examination is performed. Women of childbearing potential undergo a urine pregnancy test prior to each vaccination. A blood sample is drawn prior to immunization for HPV antibody assays. Following randomization, the first dose is administered, and participants are observed for 15 minutes following vaccination. Participants are provided with a diary to record any event for the 5 days following each dose (Table 2).

#### Follow-up Visits

A month after completion of 3 vaccinations (at study month 7), participants return for follow-up evaluation, blood draw, and optional oral sample. At study months 18 and 36, anal or vulvar swabs are obtained, and HRA or vulvoscopy is performed as surveillance for HSIL recurrence. If a lesion suspicious for HSIL is identified during the month 18 visit, a biopsy is taken. All participants receive a biopsy of any visible suspicious lesion at month 36, or at the site of the qualifying lesion if no lesion is identified. If HSIL is identified, the participants are asked to follow up with their health care clinician. Those who have a recurrence identified continue on the protocol to complete data collection for a secondary end point.

Telephone interviews are conducted at months 12, 24, and 42; they include questions about general health and anogenital pathology. Pathology and treatment information for any anogenital biopsies conducted outside the study are retrieved and abstracted for the study (Table 2).

#### Randomization

**Figure 2.**
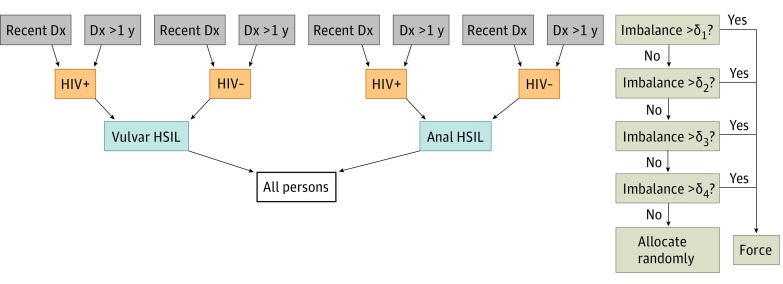
Allocation Algorithm for the Vaccine to Interrupt Progression of Vulvar and Anal Neoplasia Trial Using Dynamic Randomization The procedure for randomizing a new participant is as follows. At the top level, within all those persons sharing the same anatomical site, HIV status, and category of time since diagnosis as the current participant to be enrolled, we check whether there are approximately equal numbers randomized to both groups. If strong imbalance exists (δ_1_ > 3, where δ is the difference between numbers enrolled to placebo and vaccine), then we force that participant into the group with fewer participants to improve the balance of characteristics between the vaccine and placebo groups. If no strong imbalance exists, we proceed to check the balance at the second level of hierarchy, and so on. We set δ_1_ = δ_2_ = δ_3_ = δ_4_ = 3. Dx indicates diagnosis; HSIL, high-grade squamous intraepithelial lesions.

Enrolled participants are assigned at random to the 9vHPV vaccine group or the placebo group in a 1:1 ratio on the day of study enrollment. We conduct dynamic randomization^37^ designed to be balanced by the following strata, in order of importance: anatomic site of lesion (vulva or anus), HIV status (positive or negative), and time since HSIL diagnosis (<1 year or ≥1 year) (Figure 2). In brief, participants are allocated randomly in a 1:1 fashion to vaccine or placebo unless there exists a moderate imbalance in enrollment among persons accrued to date, by group, within levels of the strata described above. In simulation, this design forces fewer than 15% of participants into a determined group, with the rest allocated without regard to the allocations of past participants. This low number of deterministic assignments compares well with Signorini et al,^37^ who expected approximately 10% of allocations to be forced when assigning 200 participants into 2 to 3 strata. By imposing balance within the strata, the procedure ensures maximum power to detect differences in vaccine efficacy overall and by these strata (eg, we may find that vaccine efficacy is stronger in patients not infected with HIV). Both participants and study staff involved in evaluation of participants are blinded to vaccine or placebo allocation.

#### Sample Size Calculation

The sample size was determined by assuming 30% incidence of HSIL recurrence over 36 months among unvaccinated participants. We also assumed that vaccination with 9vHPV vaccine reduces the hazard of recurrence by 50%, for a cumulative incidence rate of 16% over 36 months among those who receive the active vaccine. Approximately 310 participants are needed to achieve 80% power given 1:1 randomization to vaccine and placebo. In prior studies conducted by our group, the loss to follow-up over 1 year is less than 3%. Because this study involves 36 months of follow-up, we will enroll 345 participants to allow for approximately 10% loss to follow-up.

### Statistical Analysis Plan

#### Data Management

We anticipate some participants will miss visits, which adds uncertainty to the timing of events. For those who have a recurrence detected following 1 or more missed visits, a sensitivity analysis will be performed setting the timing of the recurrence first to its detection date and second to the date of the missed visit prior to detection. Similar to interval-censored methods, and in the setting of moderately stable hazards, we expect that exact timing is not critical to determine a difference in hazard of event by group.^38^ Other violations, such as failure to complete all vaccine doses, will be handled by comparison of the intention-to-treat (ITT) and per-protocol (PP) analyses.

#### Analysis Populations

The ITT population will be all participants who have been randomized and received a dose of the vaccine. The PP population will be those who received 3 doses of the vaccine and did not have a subsequent HSIL within 1 month of the last dose. These persons in the PP population will also be required to have an HPV DNA type detected in the qualifying lesion that corresponds to HPV types included in the vaccine. Efficacy analyses will be conducted on both the ITT and PP populations.

### Primary End Point: First HSIL Recurrence

We will evaluate differences in the hazard of recurrence using Cox proportional hazards in the ITT population and the PP population if the assumption of proportional hazards is not violated; otherwise, we will use log-rank methods for crossing hazards.^39,40^ As this is a randomized trial, the primary analysis will include no other potential predictors of recurrence except for the treatment group: vaccine vs placebo. However, additional known risk factors will be included in secondary analyses of the primary aim, to confirm findings and to adjust for potential imbalances during randomization.

#### Influence of Known Risk Factors

In secondary analyses, we will adjust for the stratification factors of anatomic site, HIV status, and time since diagnosis of the qualifying lesion, as their inclusion may increase power.^41,42^ We will also assess balance with regard to smoking status and include it in secondary models. We will assess whether to include time-varying smoking as a binary covariate using current consumption levels among smokers. Graphical depictions of end point rates by levels of smoking can help determine appropriate parameterization.

#### Influence of Viral Persistence

We will evaluate whether HSIL recurrence is more frequent among those with HPV persistence, defined as the detection of the same HPV DNA type or sequence variant in swab samples collected at months 0, 18, and 36. A Cox proportional hazards approach will include a time-dependent covariate for HPV persistence. Here, we will include vaccination status in the model and include HPV persistence as both a main effect and an interaction term. This will allow us to determine whether the presence of HPV genotype or HPV-16 sequence variant impacts the risk of HSIL recurrence differently depending on vaccination receipt.

#### Influence of HPV Antibody

We will assess whether presence and amount of HPV antibody, detected at baseline in the placebo group, is protective against HSIL recurrence, separately among vaccine and placebo recipients. Subsequent graphical analysis examining incidence of recurrence by quartiles and deciles of antibody level will allow visual assessment of the relationship between levels of antibody and risk of recurrence. In further exploratory analyses, for the vaccine group, we will assess whether magnitude of vaccine-induced antibody levels a month following the third vaccination in the vaccine group is higher among those with detectable natural antibody at month 0 vs those without and whether prior antibody additionally reduces risk of recurrence. Should a threshold be observed, we may be able to determine the level of antibody associated with protection.

### Secondary End Point: Frequency of HSIL Recurrence

We will compare the frequency of HSIL recurrence among those vaccinated vs those who received the placebo during follow-up for up to 36 months. In contrast to other aims, this analysis will include all recurrences, not just first recurrences. Thus, the time following first recurrence (primary end point) will be included as time at risk for additional recurrences. We will use Poisson regression with a robust variance estimate, comparing the total number of recurrences in each group with the total follow-up per group. This analysis is included in part to mitigate the potential influence of early recurrences, which may occur prior to the full benefit of the vaccine being achieved.

## Discussion

The VIVA trial will address the proof-of-concept question for the therapeutic use of the 9vHPV vaccine among persons with HPV-associated anal or vulvar HSIL. If successful, this trial could lead to changes in the management of HSIL. The administration of the licensed HPV vaccine would be a low-cost adjunctive treatment for anal and vulvar HSIL that may reduce the frequency of surveillance and retreatment. Trial results may also clarify the role of natural antibodies in patients with recurrent HSIL.

Observational studies suggest that the HPV vaccine may provide a partial therapeutic effect in already infected patients.^28,29,30^ For example, in a pooled analysis^28^ of findings from the 4vHPV vaccine licensure trials, 587 4vHPV vaccine and 763 placebo recipients who underwent surgical treatment of squamous intraepithelial neoplasia of the cervix had 50% reduction (incidence of 6.6 and 12.2 in vaccine and placebo groups, respectively) in recurrence of cervical disease among vaccinated women. In a retrospective study^30^ of 88 men with anal HSIL, those who received the 4vHPV vaccine after treatment had a 50% reduction of HSIL recurrence compared with unvaccinated men. Another retrospective study^21^ of 65 women with VIN3 examined the recurrence of vulvar HSIL and found that women who mounted natural antibodies to HPV-16 had a 30% decreased risk of recurrent lesions. These nonrandomized studies suggest that the immune response after natural infection and 4vHPV vaccination reduce the risk of HSIL recurrence. In contrast, a randomized trial^43^ conducted to assess whether 4vHPV vaccine prevents new HPV infections or decreases anal HSIL recurrence that enrolled 472 HIV-infected men and 103 HIV-infected women in the United States was stopped early for futility. After a median follow-up of 3.4 years, vaccine efficacy was 22% (95% CI, −31% to 53%) for prevention of persistent infection. That study also failed to find evidence supporting an adjunctive role for HPV vaccination to improve outcomes following treatment of anal HSIL. Early study termination and lower than expected anal HPV infection affected the study’s ability to show efficacy.

Additional clinical trials to address the use of the HPV vaccine among infected persons are ongoing. In South Africa, a randomized trial will assess the impact of the 4vHPV vaccine against recurrence of cervical HSIL (NCT01928225) among 180 HIV-infected women followed up for 1 year; the relatively short follow-up duration and lower recurrence of cervical HSIL after treatment may limit the number of events detected. Another randomized trial (NCT02087384) in the Netherlands will assess the cumulative recurrence of anal lesions among HIV-infected MSM receiving the 4vHPV vaccine. Unlike the VIVA trial, these complementary trials are conducted exclusively in HIV-infected persons. Our target population is primarily identified from a population-based cancer registry and includes HIV-negative and HIV-positive persons with anal or vulvar HSIL.

Strengths of this trial design include recruitment of participants for a vaccine study through a population-based cancer registry, central pathology review for histologic samples to provide precision to study end points, and use of dynamic randomization to maximize power overall and within strata. While we could have also conducted block randomization within strata, Signorini et al^37^ have shown that major imbalances are possible when strata define relatively small groups. Imbalance can lead to bias, while maximizing balance also optimizes power.

### Limitations

There are some limitations to our study design. The study population has the potential to be heterogeneous (eg, women, men, HIV positive, and HIV negative), and dysplasia progression may vary between these groups. Additional studies may be necessary to determine vaccine efficacy between individual strata. Because the ultimate goal in the treatment of HSIL is to prevent the development of invasive cancer, the direct effect of the 9vHPV vaccine on cancer incidence will not be evaluated. However, we have defined a target population with sufficiently high recurrence burden to definitively assess the primary end point, which is an accepted surrogate for anal and vulvar cancer. Additional pragmatic trials may be needed to evaluate vaccine timing and implementation as an adjuvant therapy in clinical practice, particularly in low-resource areas. Integration of vaccine implementation will face several obstacles, especially in finding and treating patients who are treated for HSIL. Currently, there is a lack of screening and treatment guidelines for patients with noncervical HSIL disease, and health professionals need to be trained to appropriately evaluate these patients.

Secondary prevention of HPV-related anal and vulvar HSIL following treatment represents a medical need, particularly for at-risk populations such as MSM, HIV-infected persons, and solid-organ transplant recipients.^44,45,46,47^ Recurrences have been associated with decreased quality of life and significant morbidity due to disfiguring tissue removal and loss of function.^7,48^ In addition, follow-up and treatment of HSIL can be anxiety provoking and expensive. We estimated the potential cost of this intervention based on preliminary data assumptions and current vaccine cost: anal and vulvar recurrence rate of 30%, reduction of recurrences of 50% in vaccinated persons, and vaccine series cost of $375. The number needed to treat is 6.7; thus, anal or vulvar HSIL recurrence can be prevented for $2513. If this therapeutic approach is proven successful, immunization with 9vHPV vaccine may be a cost-effective approach to reducing the burden of recurrent HPV-related lesions.

## Conclusions

We designed this clinical trial to evaluate the ability of a highly effective and safe prophylactic HPV vaccine in the prevention of secondary anal and vulvar HSIL. The study targets adults aged 27 to 69 years with a relatively uncommon precancerous lesion but with high rates of recurrence after primary treatment. Trial findings have the potential to provide critical knowledge in the possible use of the 9vHPV vaccine as an adjuvant therapy to current treatment regimens for vulvar and anal HSIL. If this study finds that this low-cost intervention is safe and successful, further research could be extended to other anatomical sites and populations in addition to evaluating the duration of protection against recurrent HSIL.

## Notes

Supplement. **Trial Protocol**
