# Supplementary material for: Effect of Human Papillomavirus Vaccine to Interrupt Recurrence of Vulvar and Anal Neoplasia (VIVA): A Trial Protocol
Source: JAMA Netw Open. 2019 Apr 12;2(4):e190819. doi: 10.1001/jamanetworkopen.2019.0819 (PMC6481452; doi:10.1001/jamanetworkopen.2019.0819)
Supplement: Supplement. — Trial Protocol [file jamanetwopen-2-e190819-s001.pdf]

HPV Vaccine to Interrupt Progression of Vulvar and Anal Neoplasia (VIVA) Trial: A Randomized, Double-Blind, Placebo-Controlled Trial

Study Protocol

Version 7.0
26 July 2018

Principal Investigators: Margaret M. Madeleine, PhD
Fred Hutchinson Cancer Research Center
1100 Fairview Ave N., PO Box 19024
Seattle, WA 98109
Telephone: (206) 667-4629
Email: mmadelei@fhcrc.org

Anna Wald, MD, MPH, University of Washington
Harborview Medical Center, 325 9th Ave
Virology Research Clinic, Box 359928
Seattle, WA 98104
Telephone: (206) 520-4340
Email: annawald@uw.edu

Clinical Co-Investigators: Constance Mao, MD
Jeffrey Schouten, MD
Verena Grieco, MD

Statistician: Amalia Magaret, PhD, University of Washington
Harborview Medical Center, 325 9th Ave
Virology Research Clinic, Box 359928
Seattle, WA 98104

Study Site: University of Washington Virology Research Clinic
908 Jefferson St.
Suite 11NJ-1166
Seattle, WA 98104

TABLE OF CONTENTS

PROTOCOL SUMMARY 6

GLOSSARY OF TERMS 7

LIST OF ABBREVIATIONS 9

1 BACKGROUND 11

1.1 Burden of Disease 11
1.2 Prior evidence of safety 11

2 HYPOTHESIS, STUDY OBJECTIVE, AND OUTCOMES 12

2.1 Hypothesis 12
2.2 Study Objective 12
2.3 Outcomes 12
2.3.1 Primary Endpoint 12
2.3.2 Secondary Endpoints 12

3 STUDY DESIGN 12

4 PARTICIPANT SELECTION 13

4.1 Inclusion Criteria 13
4.2 Exclusion Criteria 13
4.3 Recruitment Process 13

5 STUDY PROCEDURES 14

5.1 Screening Visit (Visit -1) 14
5.2 Enrollment Visit (Month 0) 15
5.3 Months 2 and 6 15
5.4 Month 7 16
5.5 Months 18 and 36 16
5.6 Months 12, 24 and 42 16
5.7 Reminder Calls 16
5.8 Dual Site Lesions 16
5.9 Randomization and Blinding 17

6 POTENTIAL RISKS & BENEFITS 17

6.1 Potential Risks 17
6.2 Potential Benefits 19

7 CLINICAL PROCEDURES 19

7.1 Blood Collection 19
7.2 HPV Swab Collection 19
7.3 HPV Oral Rinse Collection 20
7.4 Data Collection 20
7.5 High Resolution Anoscopy 20
7.6 Anal Biopsy 20
7.7 Vulvoscopy 21
7.8 Vulvar Biopsy 21

8 LABORATORY PROCEDURES 21

8.1 Urine Pregnancy Testing 21
8.2 HIV-1 Antibody Testing 21
8.3 HPV Capsid Antibody Analysis 21

8.4 HPV TYPING FROM TISSUE 21
8.5 HPV TYPING FROM ORAL SAMPLES 22
8.6 FUTURE GENETIC RESEARCH 22

9 STUDY PRODUCT 22

9.1 TREATMENT GROUPS 22
9.2 STUDY PRODUCT AND SOURCE 22
9.3 STORAGE 22
9.4 RECOMMENDED DOSE AND SCHEDULE 22
9.5 ADMINISTRATION 23
9.6 CONTRAINDICATIONS 23
9.7 BLINDING 23
9.8 STUDY PRODUCT ACCOUNTABILITY 23

10 ASSESSMENT OF SAFETY 23

10.1 ADVERSE EVENTS 23
10.2 DEFINITION OF ADVERSE EVENT 23
10.3 DEFINITION OF SERIOUS ADVERSE EVENT (SAE) 24
10.4 ADVERSE EVENT REPORTING 25
10.4.1 SAE Reporting 25
10.4.2 Follow-up of SAE 26
10.5 SAFETY MONITORING 26
10.5.1 Data and Safety Monitoring Plan 26
10.5.2 Data and Safety Monitoring Board 26

11 CLINICAL MANAGEMENT 27

11.1 PARTICIPANT WITHDRAWAL OR DISCONTINUATION 27

12 DATA MANAGEMENT 27

12.1 DATA COLLECTION 27
12.2 CASE REPORT FORMS (CRF) 28

13 HUMAN SUBJECTS PROTECTIONS 28

13.1 IRB APPROVAL 28
13.2 INFORMED CONSENT 28
13.3 PROTECTION AGAINST RISK 28

14 STUDY DESIGN DETAILS 29

14.1 SAMPLE SIZE 29
14.2 RANDOMIZATION 29

15 STATISTICAL CONSIDERATIONS 30

15.1 MISSING DATA AND LOSS TO FOLLOW-UP 30
15.2 VISIT WINDOWS 30

16 STATISTICAL ANALYSIS PLAN 30

16.1 DATA HANDLING 30
16.2 ANALYSIS POPULATIONS 30
16.3 PRIMARY ENDPOINT 31
16.4 SECONDARY ENDPOINTS 31
16.5 DEPARTURES FROM MODEL ASSUMPTIONS 32
16.6 LEFT TRUNCATION 32

17 REFERENCES 33
APPENDIX A: STUDY VISIT WINDOWS 35
APPENDIX B: TABLE OF PROCEDURES 36

PROTOCOL SUMMARY

Design: Randomized, double-blind, placebo-controlled clinical trial

Population: Participants with anal (men and women with AIN2/3) or vulvar (women with VIN2/3) high-grade lesions, collectively called high-grade squamous intraepithelial lesions (**HSIL**)

Aim: To measure time to subsequent HSIL (in months) among vaccine compared with placebo recipients

Sub-Aims: 1) To assess whether HPV persistence (PCR detection of the same HPV type, or HPV16 variant, in ≥ 2 consecutive samples) is associated with risk of recurrence

2) To investigate the role of antibodies in deterring recurrence in vaccinated and naturally infected persons

3) To investigate whether the rate of recurrence is lower in vaccinated *vs.* un-vaccinated persons

Sample Size: 345 participants

Study Site: University of Washington
Virology Research Clinic
Seattle, WA

Study Duration: Clinic visits through Month 36 and telephone follow-up through Month 42

We will conduct a randomized, double-blind, placebo-controlled, proof-of-concept clinical trial of Gardasil, a 9-valent HPV vaccine (**9vHPV**), administered as treatment for anal or vulvar HSIL after initial treatment. We will enroll 345 individuals with anal or vulvar HSIL, identified from the local SEER cancer registry or by physician referral. Participants will be randomized 1:1 to vaccine or placebo. This study will take place at the University of Washington (**UW**) Virology Research Clinic (**VRC**). The Screening Visit includes consent forms, an HIV test, review of the eligibility criteria, colposcopy (vulvoscopy for those with prior vulvar HSIL and anoscopy for those with prior anal HSIL) with a biopsy if clinically indicated, swab sample of colposcopy site, and oral rinse for HPV DNA detection. Eligible participants will return for an Enrollment Visit in which they will undergo a blood draw, baseline questionnaire, and then be randomized to receive the vaccine or placebo (at 0, 2, and 6 months). Follow-up visits will occur at Months 7, 18 and 36. Procedures at follow-up visits include a blood draw and oral rinse at Month 7, and a blood draw, oral rinse, and anoscopy/vulvoscopy for surveillance at Months 18 and 36. Telephone or web-based interviews will be conducted at Months 12, 24, and 42. Participants will receive an anoscopy or vulvoscopy at Months 0, 18, and 36, with biopsies as needed if a lesion is visualized at the Month 0 and 18 visits. All participants will receive anoscopy or vulvoscopy and biopsy at the Month 36 visit. Slides from tissue samples for the qualifying lesion and potential HSIL recurrence lesions collected during follow up will be evaluated centrally by the study pathologist. We hypothesize that the vaccine will reduce risk of HSIL recurrence by 50%.

GLOSSARY OF TERMS

Low grade squamous intraepithelial neoplasia (LSIL)	Based on the Lower Anogenital Squamous Terminology (LAST project), LSIL include the following:<br>• HPV effect (condyloma, koilocytosis)<br>• Mild dysplasia<br>• VIN1 /AIN1<br><br>These diagnostic terms are used to reference the same underlying condition.
High grade squamous intraepithelial neoplasia (HSIL)	Based on the LAST project, HSIL include the following:<br>• Moderate dysplasia<br>• VIN2 or AIN2, with p16 staining as needed to rule out low-grade disease<br>• Severe dysplasia<br>• Carcinoma in situ<br>• VIN3 or AIN3<br><br>These diagnostic terms are used to reference the same underlying condition.
Single site	Participant with history of histologically confirmed **initial or recurrent** anal HSIL **or** vulvar HSIL either **on or after 1/1/2014.**
Dual sites	Participant with a history of histologically confirmed initial or recurrent anal and vulvar HSIL (both sites), with at least one HSIL diagnosed on or after 1/1/2014
Study inclusion criteria in participants with history of HSIL at **single site**	Study eligibility is based on having an anal **or** vulvar **HSIL lesion** (initial or recurrence) either **on or after 1/1/2014.**
Study inclusion criteria in participants with history of HSIL at **dual sites**	Participants with history of HSIL at both sites will have only one site included in the main analysis:<br><br>1) most recently treated; or<br>2) if both treated at the same time, the lesion diagnosed closest to 1/1/2014; or<br>3) if both treated and diagnosed at the same times, flip a coin.

Qualifying lesion	Histopathologically confirmed anal or vulvar HSIL diagnosed closest to (on or after) 1/1/2014
Criteria for randomization	Randomization criteria:<br>1) Anatomic site (vulvar or anal)<br>2) HIV status (positive or negative)<br>3) **Time since diagnosis** of the qualifying lesion (< 12 months or ≥ 12 months)

LIST OF ABBREVIATIONS

AAHS	amorphous aluminum hydroxyphosphate sulfate
AE	adverse event
AIN2	anal intraepithelial neoplasia grade 2
AIN3	anal intraepithelial neoplasia grade 3
AIN2/3	anal intraepithelial neoplasia grades 2 and 3; anal HSIL
ANCHOR	Anal Cancer HSIL Outcomes Research Study (NCT02135419)
cc	cubic centimeter
CDC	Centers for Disease Control
CIN2/3	cervical intraepithelial neoplasia grades 2 and 3
CRF	case report forms
CROI	Conference on retroviruses and opportunistic infections
CRS	clinical research support
CSS	Cancer Surveillance System, the SEER cancer registry for the Puget Sound Region
CTCAE	common terminology criteria for adverse events
DSMB	Data Safety Monitoring Board
DSMC	Data and Safety Monitoring Committee
FDA	Food And Drug Administration
FHCRC	Fred Hutch Cancer Research Center
HIPAA	Health Insurance Portability And Accountability Act
HIV	human immunodeficiency virus
HPV	human papillomavirus
HRA	high resolution anoscopy
HSD	Human Subjects Division
HSIL	high-grade squamous intraepithelial lesion
ID	identification data
IRB	Institutional Review Board
ITT	intention to treat analysis
mcg	microgram
NCT	National Clinical Trial
PCR	polymerase chain reaction

PE	physical exam
PHI	protected health information
PP	per protocol analysis
RCT	randomized controlled trial
SAE	serious adverse event
SEER	Surveillance, Epidemiology, and End Results Program
SOP	Standard Operating Procedures
SRC	Scientific Review Committee
UW	University of Washington
VIN2	vulvar intraepithelial neoplasia grade 2
VIN2/3	vulvar intraepithelial neoplasia grade 2 and 3; vulvar HSIL
VIVA	Vaccine to Interrupt progression of Vulvar and Anal neoplasia trial NCT03051516
VLP	virus like particle
VRC	Virology Research Clinic
°C	degrees centigrade
4vHPV	quadrivalent human papillomavirus vaccine
9VHP	nonavalent human papillomavirus vaccine

1 BACKGROUND

1.1 Burden of Disease

In the U.S., over 10,000 cases of anal and vulvar cancer are diagnosed annually, and most are HPV related.[1] Although mortality from anal and vulvar HSIL is low, there is a very high rate of recurrence: approximately 30% recur within 5 years. Development of recurrence(s) necessitates repeated surgeries associated with reduced function.[2] We estimate a prevalence of nearly 90,000 individuals with anal or vulvar HSIL among those diagnosed in the last 10 years in the US; in addition, the number of incident cases is increasing annually at a gradual but steady rate. As the unvaccinated population ages, HSIL will also continue to increase.

We propose to conduct a randomized, double-blinded, placebo-controlled clinical trial to evaluate whether the risk of recurrence of HSIL can be reduced by vaccination with 9vHPV. These lesions have the highest likelihood of HPV-related anogenital pre-cancers to progress to cancer, are more likely than invasive lesions to respond to the vaccine, and over 90% are caused by HPV types contained in the 9vHPV vaccine.[1,3]

This trial will be among the first to measure the potential benefit of the vaccine as an adjunct to treatment for HSIL, and the first to include non-HIV seropositive individuals and women with VIN2/3. Prior studies of prophylactic HPV vaccine efficacy avoided enrolling HPV positive individuals, and attempts to pool information from the small numbers of HPV positive participants enrolled in those trials suffer from heterogeneity due to different designs, goals, and outcomes of the pooled studies.[10] Two observational studies have compared outcomes among vaccinated and unvaccinated individuals with HPV-related cancer,[8,11] and three randomized trials of HPV-related lesion recurrence are currently recruiting. In South Africa, a randomized trial will assess the impact of 4vHPV vaccine against recurrence of CIN2/3 (NCT01928225) among 180 HIV+ women followed for 1 year, which may limit the number of events detected. Another RCT (NCT02087384), is being conducted at 4 centers in the Netherlands, and will assess 4vHPV against anal HSIL recurrence among HIV positive MSM. In a third trial within the AIDS Malignancy Consortium (NCT01461096), 464 HIV-infected MSM will be randomized to 4vHPV and followed for 3 years. These complementary trials are conducted exclusively in HIV positive individuals. We anticipate that approximately 70% of trial participants in the proposed study will be HIV negative. Our target population will be primarily identified from a population-based SEER registry, and advances beyond currently ongoing trials by including HIV negative individuals and women with anal or vulvar HSIL.

Emerging data suggest that the licensed vaccine could decrease risk of recurrence of HSIL by 50%, by increasing immune response and reducing the risk of reactivation of disease.[8] However, a recent HPV trial in HIV infected individuals with no previous HPV-associated cancers was stopped early due to lack of difference between the placebo and vaccine arms with regard to prevention of new HPV infections or to improve treatment outcomes.(Wilkin et al, CROI 2016) As this study was stopped early, follow-up time was limited to assess anal HSIL risk. We recognize that the most effective use of the vaccine is before sexual debut; this is very clear. However, the potential benefits of the vaccine are to reduce recurrence of HSIL and avoid multiple and increasingly disfiguring surgeries, psychosexual trauma, and debilitated functions through relatively low cost and, as we hope to prove, safe vaccination.

1.2 Prior evidence of safety

The common reported adverse events from the licensure for 9-valent HPV vaccine trials[12,13] report injection site reaction and lightheadedness, likely resulting from pain of injection. No clinically important safety concerns have emerged from those studies or studies during implementation.[14-16] Safety of the vaccine after treatment for HSIL is unknown, but the vaccine is

likely to be safe in that population. As our study will result in off-label use, we will monitor safety by comparing type and frequency of AEs in the two study arms.

2 HYPOTHESIS, STUDY OBJECTIVE, AND OUTCOMES

2.1 Hypothesis

The main purpose of this trial is to test whether an FDA approved prophylactic HPV vaccine (9vHPV) will lead to a 50% reduction of recurrence and progression of HSIL compared to unvaccinated individuals with HSIL.

2.2 Study Objective

We will test if the 9vHPV vaccine delivered after treatment for HSIL reduces the risk of histologically confirmed recurrent neoplastic lesions (HSIL) by 50% in the vaccinated *vs.* placebo arms. We will also evaluate safety of the HPV vaccine in HSIL participants.

2.3 Outcomes

2.3.1 Primary Endpoint

The primary endpoint of the study is time to recurrence of anogenital HSIL among vaccine compared with placebo recipients.

2.3.2 Secondary Endpoints

We will assess the safety of the vaccine.

We will additionally evaluate efficacy after adjustment for baseline stratification variables and after adjustment for HPV persistence. The impact of persistence and of HPV antibody level will be evaluated separately by vaccination arm. An additional secondary endpoint is the frequency of recurrences between arms, including all subsequent recurrences observed over the three years, rather than time to first recurrence.

3 STUDY DESIGN

We will conduct a randomized, double-blind, placebo-controlled proof-of-concept clinical trial of 9vHPV administered as an adjunctive treatment for anal or vulvar HSIL. We will enroll 345 individuals, identified using either the local SEER cancer registry, to which individuals with HSIL (AIN3/VIN3) are reported, or physician referral of participants with HSIL (AIN2/VIN2) that are confirmed by a pathologist. This study will be conducted at the VRC, where all procedures and specimen collection will be performed by experienced research clinicians. The 6-7 clinic visits include a screening visit, three vaccination visits (at 0, 2, and 6 months), and three follow up visits (at 7, 18, and 36 months). Phone call follow-ups will take place at months 12, 24, and 42 months. In some participants, the screening and the enrollment visit may be combined.

4 PARTICIPANT SELECTION

4.1 Inclusion Criteria

Men and women who meet all of the following criteria are eligible for inclusion in this study:

- Age 27- 69 at enrollment
- Histologically confirmed diagnosis of initial or recurrent anal or vulvar high-grade squamous intraepithelial lesion diagnosed on or after 1/1/2014; study pathologist will use p16 staining as needed to rule out LSIL disease
- ≥ 2 months since last therapy for HSIL.
- No clinical evidence of HSIL on screening examination; if HSIL is suspected, a biopsy will be done to exclude HSIL. Patients whose screening visit reveals HSIL on biopsy, may be re-screened one time, ≥2 months after therapy.
- Resident in the area and willing to attend up to 7 clinic visits for a 36-month period at the VRC
- Sexually active women of child-bearing potential must be willing to use effective contraception through Month 7 of the study
- If HIV positive, receipt of anti-retroviral therapy continuously for at least 6 months prior to enrollment
- Ability to give informed consent
- Willingness to sign medical records release form and tissue release form

4.2 Exclusion Criteria

Men and women who meet any of the following criteria are not eligible for this study:

- Currently pregnant
- Chemotherapy (current, within the last month, or anticipated in the next 7 months)
- Prior history of invasive HPV-related anogenital cancer (cervical, vaginal, vulvar, penile, or anal cancer), or oropharyngeal cancer (base of tongue, tonsil). Prior cancer at other sites (including most of oral cavity) or larynx are not exclusions
- Unstable medical condition (e.g., another malignancy requiring treatment, malignant hypertension, poorly controlled diabetes, another cancer except for fully excised non-melanoma skin cancer)
- Prior HPV vaccination
- Known allergy or intolerance to lidocaine
- Currently participating in an interventional research study related to HPV, except the ANCHOR study (NCT02135419)
- Any other condition which, in the opinion of the investigator, may compromise the subject's ability to follow study procedures and safely complete the study.

4.3 Recruitment Process

We will recruit 345 men and women with HSIL from the greater Seattle area. Potentially eligible participants will be referred to study staff who will discuss the study and eligibility criteria with the participant. Those that are eligible and interested will arrange a screening appointment at the clinic.

Sources of potential participants include but are not limited to:

- The Cancer Surveillance System (CSS) of the Fred Hutchinson Cancer Research Center, a population-based National Cancer Institute Surveillance, Epidemiology, and End-Results (SEER) cancer registry.
- Physicians referrals
- Pathology records from nearby medical institutions
- Community recruitment

All recruitment methods and materials will be IRB approved prior to use.

We will obtain a partial waiver of HIPAA authorization and partial waiver of consent for recruitment.

5 STUDY PROCEDURES

5.1 Screening Visit (Visit -1)

After receipt of the approach letter or a brochure from their healthcare provider, interested potential participants will be screened by telephone regarding study eligibility criteria. Potentially eligible participants will schedule a Screening Visit at the VRC.

During the Screening Visit, study background and procedures will be explained. Potential participants will have a chance to ask questions about all aspects of the study. Written informed consent, HIPAA Authorization, and Medical Records and Tissue Releases will be obtained prior to participation in the study. Inclusion/exclusion criteria will be reviewed and the qualifying lesion for inclusion and randomization will be determined:

Study inclusion in participants with history of HSIL at **single site**	Study eligibility is based on having an anal **or** vulvar **HSIL lesion** (initial or recurrence) either **on or after 1/1/2014.**
Study inclusion in participants with history of HSIL at **dual sites**	Participants with history of HSIL at both sites will have only one site included in the main analysis:<br>1) most recently treated; or<br>2) if both treated at the same time, the lesion diagnosed closest to 1/1/2014; or<br>3) if both treated and diagnosed at the same times, flip a coin.

Demographic and baseline medical history information will be obtained which will include questions about general health, medical conditions, concomitant medications, and anogenital pathology including treatments. Sexually active women of childbearing potential will undergo a urine pregnancy test. Blood will be drawn for HIV testing, if needed. An oral rinse will be collected for HPV DNA detection. A general physical exam

will be performed. Anoscopy or vulvoscopy will be performed of the anus or vulva, depending on the location of the qualifying lesion. Swab samples will be collected. A biopsy will be taken if any lesions suspicious for HSIL are visualized at the qualifying site.

If a biopsy is collected during the Screening Visit:

- Participants with negative biopsy results will be eligible for enrollment into the study.
- Participants with positive biopsy results will be deferred but will be offered the ability to rescreen $\geq$2 months after treatment. The results of the biopsy will be shared with the participant and their healthcare provider. Any follow-up treatment will be provided by the outside provider, not by the study.

Individuals who are eligible to enroll in the VIVA Trial at the Screening Visit may undergo a combined Screening Visit and Enrollment Visit. Individuals in need of medical documentation before becoming eligible (i.e., negative biopsy results or confirmation of HIV status) will be asked to schedule their Enrollment Visit within 8 weeks of their Screening Visit.

Individuals who had anoscopy or vulvoscopy performed within 8 weeks of the Screening Visit by Dr. Constance Mao, Dr Jeffrey Schouten, or an ANCHOR-certified provider and had no evidence of vulvar or anal HSIL will not need anoscopy or vulvoscopy performed at the Screening Visit. They will undergo all other screening procedures. Documentation of the high resolution anoscopy or vulvoscopy procedure, cytology, and any biopsies performed will be requested and reviewed prior to entry to verify that no HSIL was detected on recent HRA or vulvoscopy exams.

5.2 Enrollment Visit (Month 0)

Inclusion/exclusion criteria will be reviewed. Interim medical history and concomitant medications will be collected. A targeted physical exam will be performed. Vital signs (blood pressure, pulse, temperature) including self-reported height and weight will be collected. Women of childbearing potential will undergo a urine pregnancy test. Up to 20 cc of blood will be drawn for HPV antibody assays.

Participants will be randomized and the study product will be administered via intramuscular injection in the deltoid region of the preferred arm. Participants will be observed for 15 minutes after vaccine dose administration. Participants will be instructed about potential adverse events (AEs), and provided with a log to record any fever or injection site reactions for the next 5 days.

Participants will complete a self-administered questionnaire.

5.3 Months 2 and 6

Interim medical history and concomitant medications will be obtained including any new anogenital biopsies or treatments. A targeted physical exam will be performed. Vital signs

(blood pressure, pulse, temperature) will be collected. Women of childbearing potential will undergo a urine pregnancy test prior to each vaccination.

The study product will be administered via intramuscular injection in the deltoid region of the preferred arm. Participants will be observed for 15 minutes after vaccine dose administration. Participants will be instructed about potential AEs, and provided with a log book to record any fever or injection site reactions over the next 5 days.

Participants will complete a self-administered questionnaire at Month 6.

5.4 Month 7

Interim medical history and concomitant medications will be obtained including any new anogenital biopsies or treatments. A targeted physical exam will be performed. An oral rinse will be collected for HPV DNA detection. Blood will be drawn (up to 30 cc serum) for HPV antibody assays and DNA (optional).

5.5 Months 18 and 36

Interim medical history and concomitant medications will be obtained including new anogenital biopsies or treatments. A targeted physical exam will be performed. Blood will be drawn (up to 20 cc serum) for HPV antibody assays. An oral rinse for HPV DNA detection will be collected. Swabs will be obtained at the Months 18 and 36 visits at the qualifying lesion site of the vulva or anus, and anoscopy or vulvoscopy will be conducted as surveillance for recurrence. A biopsy will be taken, if needed, at Month 18. All participants will receive a biopsy of any visible lesion suspicious for HSIL, or at the site of the qualifying lesion if no lesion is identified, at Month 36. The results of the biopsy(s) will be shared with the participant and their healthcare provider. Any follow-up treatment will be provided by the outside provider, not by the study.

Participants will complete a self-administered questionnaire.

5.6 Months 12, 24 and 42

Telephone interviews will be conducted at Months 12, 24 and 42. These short interviews will include questions about health, smoking status, and anogenital disease.

5.7 Reminder Calls

Reminder calls will be conducted every 1-3 months after the Month 12 visit for study retention. Reminders will be made by phone, email or text per participant preference.

5.8 Dual Site Lesions

Definition of Dual Lesions: Participant with a history of histologically confirmed initial or recurrent anal and vulvar HSIL (both sites), with at least one HSIL diagnosed on or after 1/1/2014.

Individuals with lesions at dual sites will be offered a complimentary HRA or vulvoscopy exam that can be performed at any time after participant enrollment based on participant and clinician availability.

Information about the dual site lesion history will be captured and become part of the study record. Subsequent vulvoscopy or anoscopy will be offered at 18 and 36 months, according to the single site follow up examinations.

5.9 Randomization and Blinding

Enrolled participants will be assigned at random to one of two groups in a 1:1 ratio. Both study staff involved in evaluation of participants and participants are blinded to the assignments of participants to the study groups. We will conduct dynamic randomization so that it will be balanced by the following groups, in order of importance: anatomic site of lesion, HIV status, and time since diagnosis of the qualifying lesion (<12 months, ≥ 12 months). The qualifying lesion is defined as a histopathologically confirmed anal or vulvar HSIL diagnosed closest to, but on or after, 1/1/2014. Anatomic site will largely divide participants on gender, so further stratification by gender is unnecessary. Similarly, HIV status will be related to gender, as most women will be HIV negative while many men with anal HSIL (80% in our pilot study) will be HIV positive.

Please refer to the Randomization Standard Operating Procedure (SOP) for details about the randomization process for study staff involved in randomizing participants.

Randomization documentation and other pharmacy records will be stored in a secure location in the site pharmacy apart from the rest of the participant file. This information will not be accessible to study staff members who complete other study procedures with participants. Blinding will be maintained until all data are entered into the study database, all study endpoint data and other data included in the final analysis have been verified, and the data are ready for final analysis. If unblinding is needed for provision of medical treatment or to otherwise to protect the safety of study participants, DSMB input will be sought.

At the end of the study, participants will be unblinded and provided their group assignment. Participants who received placebo will be offered the vaccine, free-of-charge.

6 POTENTIAL RISKS & BENEFITS

6.1 Potential Risks

Phlebotomy: Blood draw may cause faintness, discomfort and/or bruising. Rarely, an infection may develop.

Data Collection: Answering questions about medical history may be stressful.

Pregnancy: The safety of 9vHPV has not been established in pregnant women. Pregnant women will be excluded from the study at enrollment. Women who become pregnant will remain in the study and receive the remaining doses of the vaccine after the pregnancy resolves. Women who become pregnant after the 7th month of the study will remain in the study.

Vaccination: Safety of the vaccine after treatment for HSIL is unknown, but the vaccine is likely to be safe in this population. Since our study will result in off-label use, we will monitor safety by comparing type and frequency of AEs in the two study arms.

The most common adverse reaction in prior HPV trials was headache. Adverse reactions include fever, nausea, and dizziness; and local injection site reactions (pain, swelling, erythema, pruritus, and bruising). Syncope, sometimes associated with tonic-clonic movements and other seizure-like activity, has been reported following vaccination with 9vHPV and may result in falling with injury; observation for 15 minutes after administration is recommended. Anaphylaxis has been reported following vaccination with 9vHPV. As with any vaccination, there may be side effects that are not known at this time.

Anal Swabs: Putting a swab into the anus may cause some discomfort. Minor bleeding (less than a quarter of a teaspoon) rarely occurs in some people due to the insertion of the swabs. The bleeding stops almost immediately.

High Resolution Anoscopy: Insertion of an anoscope will likely cause some discomfort. Participants may feel pressure and the urge to have a bowel movement. Putting acetic acid (vinegar) in the anal canal rarely causes minor burning and irritation.

Anal Biopsy: Participants may have pain with the anal biopsies. Participants may have some bleeding for up to a week after biopsies, especially when they experience a bowel movement. There is a rare chance of very heavy bleeding that may require extra treatment. There is a very slight risk of infection (<1%). Participants will be instructed to contact the study clinic if they have symptoms of heavy bleeding or infection (fever, pain, redness, or swelling).

We will ask participants to refrain from receptive anal sex for 24 hours before the biopsy and to refrain from taking aspirin and ibuprofen, or medications containing these, prior to the biopsy. This will help prevent any possible increase in risk of infection or bleeding after the procedure. We will instruct participants to avoid aggressive exercise immediately after the biopsy and to not engage in receptive anal sex one week after the rectal biopsy.

Vulvoscopy: Participants may feel discomfort during the procedure. Application of acetic acid for the exam may cause a burning sensation.

Vulvar biopsy: Injection of a local anesthetic prior to the biopsy maybe painful and the biopsy itself may cause pain, even with the use of an anesthetic. Biopsies may cause a pressure or tugging sensation. Bleeding and pain or irritation at the biopsy site(s) may continue for up to a week following the procedure. Although the skin biopsies are small (3-4mm), scarring may occur at the site. Participants will have to avoid sexual contact for 5 days after a biopsy, or until healing is complete.

Lidocaine (injectable, +/- epinephrine): Lidocaine administration is associated with temporary pain and burning. Allergic reactions to lidocaine may occur, even in participants

with no history of allergic reactions. Examples of allergic reactions include wheezing or difficulty in breathing, lightheadedness or fainting, skin rash and itching. As with any drug there may be unknown side effects. Biopsies may cause discomfort even when the area is numbed using lidocaine

Silver nitrate: Silver nitrate may be used if there is excessive bleeding after a biopsy. Allergic reactions to silver nitrate may occur, even in participants with no history of allergic reactions. Examples of allergic reactions include wheezing or difficulty in breathing, lightheadedness or fainting, skin rash and itching. As with any drug there may be unknown side effects.

Monsel's Solution: Topical Monsel's solution may be used for excessive bleeding after a rectal biopsy. There are no allergic reactions or adverse effects from application of Monsel's solution.

Collection of Oral Rinse: If a sample is collected when oral lesions are present, this may cause discomfort while using the oral mouthwash.

6.2 Potential Benefits

Participants may benefit from the study by receiving the 9vHPV vaccine, otherwise, participants are not expected to benefit from participation in this trial. If this trial successfully shows that the HPV VLP vaccine is effective and provides proof for a conceptual framework, future studies could address immunogenicity over a longer time-period, and assess timing of doses with respect to HSIL diagnosis. If this trial shows that the HPV VLP vaccine is not a useful adjunctive care in this setting, future studies could determine if variant types in persistent infections or antibody assays could be employed as potential biomarkers of recurrence that could impact clinical surveillance.

7 CLINICAL PROCEDURES

7.1 Blood Collection

Blood will be obtained by routine venipuncture.

7.2 HPV Swab Collection

Dacron swabs will be used to obtain a sample at the site of the qualifying lesion of the vulva or anus, depending on location of qualifying lesion for this study. Samples will be collected at Months 0, 18, and 36.

For vulvar sampling, the swabs will be moved back and forth, in a tight zigzag motion from the clitoral prepuce down to the posterior fourchette, on one side and then the other, to allow collection on both sides of the perineum between the folds of the labia minora and majora.

Men and women with prior anal lesions will have a specimen collected by insertion of a dry swab 3–4 cm into the anal canal; the swab will be rotated once, and then removed

with rotation and continued gentle pressure against the wall of the anal canal and perianal area.

Specimens will be placed in labeled collection tubes containing transport medium, the handle snapped off at the perforation, capped, and kept frozen at -20°C until assayed.

7.3 HPV Oral Rinse Collection

For oral rinse sampling, participants will be asked to swish and deep gargle Scope solution for 30 seconds. The participant will spit the mouthwash into a specimen container. The specimen container will be capped, labeled, processed per SOP, and kept frozen at -20°C until assayed.

7.4 Data Collection

Participants will be asked questions about their health and anogenital pathology at the first and follow-up visits. Self-administered questionnaires will be completed at Enrollment, Month 6, Month 18 and Month 36. Questions will focus on general health, medications, any new biopsies or surgeries, smoking history, sexual history, mental health, and daily activities. Report of any anogenital biopsies between study visits will trigger requests for pathology reports to review for anal or vulvar HSIL recurrences at the qualifying site.

We will retrieve medical records and pathology reports describing 1) treatment for AIN2/3-VIN2/3 (defined as the qualifying lesion, although the individual may have had other prior AIN2/3-VIN2/3 diagnosis prior to the qualifying diagnosis; and reports of 2) clinical and histopathological diagnosis of anal or vulvar HSIL that was done outside the study visits to confirm recurrence of HSIL. The qualifying lesion and biopsies taken during follow up will be centrally reviewed by the study pathologist.

If HRA or vulvoscopy evaluation was done at the site other than the qualifying lesion, this information will be collected and will become part of the study record.

7.5 High Resolution Anoscopy

A digital anal/rectal exam will be performed. A lubricated plastic anoscope will be inserted into the anus. Then, a piece of gauze moistened with acetic acid is placed in the anus for 1-2 minutes to enhance visualization of abnormal areas. The anoscope will be put back into the anus. A colposcope is then used to visualize the skin inside the anus. Iodine may also be used to enhance visualization of lesions.

7.6 Anal Biopsy

The anal canal will be visualized during anoscopy. Topical lidocaine lubricant will be used to anesthetize the area, and lidocaine or bupivacaine may be injected if the lesion is near the anal verge on the perianal skin. A biopsy of an anal canal lesion is 2-3 mm.

7.7 Vulvoscopy

In dorsal lithotomy position, the entire vulva, mons, and perianal area will be inspected for lesions. 5% acetic acid will be applied liberally to the entire area for 3- 5 minutes. Careful examination will be performed with a colposcope and any suspicious lesions will be identified by location and clinical impression.

7.8 Vulvar Biopsy

The vulva will be prepped with chlorhexidine or Betadine solution. Approximately 1cc of 1% lidocaine (+/- epinephrine) will be injected to anesthetize the area for biopsy. 3 or 4 mm punch biopsy will be used to take biopsy. Silver nitrate may be used as a cautery agent to control bleeding.

8 LABORATORY PROCEDURES

Laboratory procedures include HIV testing, HPV DNA typing and variant sequencing, and HPV serology. Centralized pathology review of qualifying lesion tissue samples and potential recurrences will be reviewed at the UW (by Dr. V. Grieco). Future studies using samples collected in this study will need to be approved by the Institutional Review Board.

Assays may also be conducted with researchers from other universities and from pharmaceutical companies.

8.1 Urine Pregnancy Testing

Urine pregnancy testing will be performed per manufacturer's guidelines.

8.2 HIV-1 Antibody Testing

HIV-1 antibody testing will be performed at Screening Visit, as needed. HIV-1 testing will be repeated if previous testing was performed > 12 months prior to the Screening visit (> 6 months for high risk participants).

8.3 HPV Capsid Antibody Analysis

Serum will be drawn into a red-top tube, processed and delivered to the laboratory for HPV capsid antibody assays.

8.4 HPV Typing from Tissue

Investigators will retrieve tissue associated with the qualifying lesion and recurrent diagnoses from study clinicians or, if between visits, local providers. All tissue blocks will be reviewed and sectioned for HPV DNA testing by Dr. Grieco. We will perform HPV typing and HPV16 lineage assessment on HPV16 positive samples.

8.5 HPV Typing from Oral Samples

Oral rinse specimens will be collected at Screening, and Months 7, 18, and 36 for type-specific HPV using polymerase chain reaction (PCR)-based method according to the protocol recommended by the manufacturer.

8.6 Future Genetic Research

Samples will be collected for future genetic studies of HPV-related conditions using DNA from blood collected at Month 7. All such studies will be performed by the study Investigators or their scientific collaborators and will be reviewed and approved by the Institutional Review Board.

9 STUDY PRODUCT

9.1 Treatment Groups

Name	Dose	How administered
Gardasil®	0.5 ml dose	Intramuscular injection in the deltoid region of the arm
Placebo	0.5 ml saline	Intramuscular injection in the deltoid region of the arm

9.2 Study Product and Source

Gardasil: Pre-filled vials with vaccine (Gardasil, 9vHPV) will be obtained from Merck. The 9vHPV vaccine is FDA approved.

Placebo: Normal saline will be obtained from a commercial manufacturer.

9.3 Storage

9vHPV: The vaccine will be stored at 2 to 8°C (36 to 46°F) and protected from light until use. The vaccine will be administered as soon as possible after being removed from refrigeration.

Placebo: The placebo will be stored per package guidelines.

9.4 Recommended Dose and Schedule

Each 0.5-mL dose of 9vHPV contains approximately 30 mcg of HPV Type 6 L1 protein, 40 mcg of HPV Type 11 L1 protein, 60 mcg of HPV Type 16 L1 protein, 40 mcg of HPV Type 18 L1 protein, 20 mcg of HPV Type 31 L1 protein, 20 mcg of HPV Type 33 L1 protein, 20 mcg of HPV Type 45 L1 protein, 20 mcg of HPV Type 52 L1 protein, and 20 mcg of HPV Type 58 L1 protein. Each 0.5-mL dose of the vaccine also contains approximately 500 mcg of aluminum (provided as AAHS), 9.56 mg of sodium chloride, 0.78 mg of L-histidine,

50 mcg of polysorbate 80, 35 mcg of sodium borate. The product does not contain a preservative or antibiotics.

Participants will be administered vaccine at 0, 2, and 6 months, as per the 9vHPV package insert. The preferred injection site is the deltoid muscle, preferably in the non-dominant arm, with intramuscular deposition.

9.5 Administration

9vHPV should be thoroughly shaken before use and administered as soon as possible after being removed from refrigeration. After thorough agitation, 9vHPV is a white, cloudy liquid. Parenteral drug products should be inspected visually for particulate matter and discoloration prior to administration. The product should not be used if particulates are present or if it appears discolored.

The placebo (0.5 ml saline) will be prepared by the site pharmacist or clinician per package guidelines.

All participants will be observed for syncope for 15 minutes after vaccine dose administration, as syncope has been reported following vaccination with 9vHPV. The CDC Vaccine Information Sheet will be given with each vaccination to the participant.

9.6 Contraindications

Hypersensitivity, including severe allergic reactions to yeast (a vaccine component).

9.7 Blinding

An unblinded research pharmacist or clinician will administer the immunization to maintain the blind, as per clinic standard procedures.

9.8 Study Product Accountability

The research staff will maintain complete records of all study products received and subsequently dispensed.

10 ASSESSMENT OF SAFETY

10.1 Adverse Events

The investigator is responsible for reporting Adverse Events (AEs) that are observed or reported during the study.

10.2 Definition of Adverse Event

An adverse event (AE) is defined as any untoward medical occurrence in a participant including any abnormal sign (e.g., abnormal physical exam), symptom, or disease, temporally associated with the subject's participation in the research, which does not necessarily have a causal relationship with the study. Mechanisms of obtaining information on AE will rely primarily on visits to the clinic with a health care provider.

The occurrence and severity of all AEs will be listed and graded according to the FDA criteria (FDA Guidance for Industry: Toxicity Grading Scale for Healthy Adult and Adolescent Volunteers Enrolled in Preventive Vaccine Clinical Trials). The following information will be collected on all AEs experienced during this study:

- Name of the event: If the event is described in the FDA Toxicity Grading Scale, the Investigator should use that terminology. Otherwise, terminology that clearly describes the pathophysiology of the event and body system affected should be used.
- Onset Date
- Date of resolution
- Severity
    - o If an event is not described in the FDA Toxicity Grading Scale and therefore not graded, the following grading will be used:
        1. Mild, easily tolerated by the participant, causing minimal discomfort and not interfering with everyday activities.
        2. Moderate, sufficiently discomforting to interfere with normal everyday activities
        3. Severe, prevents normal everyday activities
        4. Life-threatening, places the participant at immediate risk for death
- Relationship to study drug: All AEs will have a causality assessment performed at the time of reporting the event to document the Investigator's perception of causality. For the purposes of this study, causality will be assigned using the following criteria:
    - o Related: The event cannot be attributed to the participant's underlying medical condition or other concomitant therapy and there is a compelling temporal association between the onset of the events and study drug administration that leads the Investigator to believe that there is reasonable chance of a causal relationship.
    - o Remote: A relationship is not obvious but cannot be ruled out.
    - o Not related: The participant's underlying medical condition or concomitant therapy can easily be identified as the cause of the event and there is no temporal relationship between the event and the study drug.

10.3 Definition of Serious Adverse Event (SAE)

The Investigator is required to determine if each AE was an SAE. An SAE is any AE occurring at any administration of study drug that results in any of the following outcomes:

- Death
- A life-threatening adverse drug experience
- A congenital anomaly or birth defect
- Inpatient hospitalization (hospitalizations for elective medical/surgical procedures, scheduled treatments, or routine check-ups are not SAE by these criteria)
- A persistent or significant disability / incapacity

Important medical events that may not meet any of the above criteria may be considered an SAE when, based upon appropriate medical judgment, it may jeopardize the participant and may require medical or surgical intervention to prevent one of the outcomes listed above.

10.4 Adverse Event Reporting

The following AEs will be collected during the study on appropriate source documentation:

- Vaccine-related AEs will be collected
- Reactogenicity will be collected though Day 5 after each dose.
- Biopsy-related AEs will be collected until healing after each biopsy.
- SAEs will be recorded until the final study visit.

Adverse Events that will be recorded in the study database are reactogenicity and any Grade 3 or higher AEs that are deemed related or possibly related to the study. All Serious Adverse Events (SAEs) will be recorded.

Please refer to the AE Documentation Standard Operating Procedure (SOP) for details about the AE documentation for study staff involved in clinical evaluation of the participants.

Information to be collected includes event description, date of onset, investigator assessment of severity, investigator assessment of relationship to study product, date of resolution of the event, seriousness, and outcome. The **intensity** of nonserious AEs can be assessed by a licensed clinician (i.e., physician, nurse, Nurse Practitioner, Physician Assistant). **Causality** of nonserious AEs can be assessed only by a clinician licensed to make medical diagnoses (ie, physician, Nurse Practitioner, Physician Assistant). All AEs will be followed to adequate resolution or until considered stable.

Any medical condition that is present at screening will be considered as baseline and will not be reported as an AE. If the severity of any pre-existing medical condition increases during the study period, then it will be recorded as an AE.

10.4.1 SAE Reporting

All SAEs will be reported immediately to Dr. Wald.

SAE Alternate: A clinician from the Virology Research Clinic is available at any time by calling the 24-hour emergency pager at 206-598-0924 and asking for the Virology Research Clinic Clinician on-call.

All SAEs will be recorded on the appropriate SAE Report Form. The SAE report will include the following information (as available):

- Participant ID
- Description of SAE (onset date, severity, causal relationship)
- Basic demographic information
- Outcomes attributed to the event

- Summary of relevant test results, laboratory data, and other relevant history
- The first and last dates of study drug administration
- Statement of whether study drug was discontinued or schedule modified
- Statement of whether the event abated after study drug was discontinued or schedule modified
- Statement of whether the event recurred after reintroduction of the study drug if it had been discontinued.

All SAEs reported to the Virology Research Clinic will be relayed to the Human Subjects Division (HSD) of the University of Washington as required by HSD policy.

10.4.2 Follow-up of SAE

All SAEs will be followed through resolution by a study physician.

10.5 Safety Monitoring

10.5.1 Data and Safety Monitoring Plan

Institutional support of trial monitoring will be in accordance with the FHCRC/University of Washington Cancer Consortium Institutional Data and Safety Monitoring Plan. Under the provisions of this plan, FHCRC Clinical Research Support (CRS) coordinates data and compliance monitoring conducted by consultants, contract research organizations, or FHCRC employees unaffiliated with the conduct of the study. Independent monitoring visits occur at specified intervals determined by the assessed risk level of the study and the findings of previous visits per the institutional DSMP.

In addition, protocols are reviewed at least annually and as needed by the Consortium Data and Safety Monitoring Committee (DSMC), FHCRC Scientific Review Committee (SRC) and the FHCRC/University of Washington Cancer Consortium Institutional Review Board (IRB). The review committees evaluate accrual, adverse events, stopping rules, and adherence to the applicable data and safety monitoring plan for studies actively enrolling or treating subjects. The IRB reviews the study progress and safety information to assess continued acceptability of the risk-benefit ratio for human subjects. Approval of committees as applicable is necessary to continue the study.

The trial will comply with the standard guidelines set forth by these regulatory committees and other institutional, state and federal guidelines.

10.5.2 Data and Safety Monitoring Board

We will convene a Data Safety Monitoring Board (DSMB) for this study. DSMB members will be recruited from among HPV vaccine and oncology experts in the Seattle area (chair and 3 members); one of the four members will be the lead biostatistician for the trial and the biostatistician for the DSMB, Amalia Magaret, PhD and there will also be an independent statistician participating. Prior to any participant enrollment in the study,

DMSB members will form an understanding of the protocol and definitions being used, and review and approve the charter.

The DSMB will conduct two independent reviews of the interim safety data, the first after the initial 50 participants complete two immunizations and the second after 150 participants complete their Month 6 Visit. In addition, any grade 3 or higher AEs that occur in more than 10% of the participants, except for local and systemic immunogenicity, will be reviewed by the DSMB chair who will then consult the remaining members. The DSMB will review data upon their request as specified in the charter.

11 CLINICAL MANAGEMENT

The clinic visits for this study will be conducted over a 36-month period for each participant. During this period, participants will be seen at clinic for injections, surveillance and study sample collection. Participants will be asked to complete one final phone call visit at Month 42.

11.1 Participant Withdrawal or Discontinuation

Normal study completion will be defined as completion of the 36-months of clinic visits plus an additional telephone interview at 42 months (or mid-point between the end of the clinic visits and the end of data collection for the study). Participants who do not appear for scheduled visits will be traced using originally provided contact information. Efforts will be made to accommodate participants who are willing to return to clinic outside of scheduled visit windows in order to maximize ascertainment.

Participants may voluntarily withdraw from the study for any reason at any time. The investigators may also withdraw participants from the study at any time to protect patient safety, if the patient is unwilling or unable to comply with required study procedures, for administrative reasons, at the request of government or regulatory agencies, or if the study is terminated early. Study staff members will record the reason(s) for withdrawal in participants' study records.

12 DATA MANAGEMENT

12.1 Data Collection

Data collection will involve questionnaires, specimen collection, and results of laboratory testing that are obtained specifically for research purposes. All participants are tracked though a unique study identification number. This number is the only unique identifier that will appear on the participant questionnaires, specimens and the databases into which these data will be entered.

Research records for participants in this study are the responsibility of the investigators. All study documents will be confidential.

Tracking data, which contains PHI, is password protected and stored on secure servers. Paper files, such as interviewer contact sheets with PHI and study identifiers, are stored separately in locked file cabinets when not in use.

12.2 Case Report Forms (CRF)

Case Report Forms will be identified with a study number. CRFs are to be completed for each participant by the investigator or designated member of the study staff and initialed by the investigator or designee. CRFs will be completed neatly and legibly. Any modification of previously entered data must be made by the investigator or designee by striking through the original entry with a single line, initialing and dating changes, and entering the correct data nearby. A valid explanation must be given for any missing information. All data will be entered into a computer database and a further quality control check will be made to produce a final database for analysis.

13 HUMAN SUBJECTS PROTECTIONS

13.1 IRB Approval

Prior to the initiation of the study, the principal investigator will obtain written approval to conduct the study from the University of Washington Human Subjects Review Committee.

13.2 Informed Consent

The investigator or study coordinator will explain the purpose and nature of the study, including potential benefits and risks to the participant, to each potential participant before enrollment in the study. Non-English readers will be consented using the short-form consent process with a qualified interpreter.

The participant must sign an informed consent form approved by the IRB before entering the study. All original informed consent forms will be retained by the Principal Investigator separately from the participant's records, as they have names. The participant will receive a copy of the signed informed consent form.

13.3 Protection against Risk

All procedures are conducted in a clinical setting with experienced research clinicians and coordinators. Information and samples are coded to protect the confidentiality of the participants and no identifying information of any kind is released to any other person or agency without specific written permission. Only the clinic staff will have access to potentially identifiable personal information. All research staff is skilled at maintaining confidentiality of the participants and their study results. All data are coded and files are maintained in locked cabinets.

We have established Standard Operating Procedures to minimize the risk of study procedures. Care will be taken during the consenting process and throughout the study to assure that participants are fully informed of all study procedures and associated risks.

Participants will also be educated about how to contact the study investigators if any questions or concerns arise.

14 STUDY DESIGN DETAILS

14.1 Sample size

The sample size will be 310, with a plan to enroll 345 to allow for 10% dropout. Our samples size was determined by assuming 30% incidence of recurrence over 3 years among those not vaccinated, and assuming that vaccination with HPV vaccine reduces the hazard of recurrence by 50%, for a vaccinated cumulative incidence rate of 16% over 3 years. We used the following formula by Lachin[17]:

$$2N = 2(Z_\alpha + Z_\beta)^2[\varphi(\lambda_C) + \varphi(\lambda_I)]/(\lambda_C - \lambda_I)^2 \qquad (1)$$

where α = type I error of 5%, β = type II error of 20% (for 80% power), λ_T is the incidence rate for each unit of time on arm T (T is either C=control, placebo or I=intervention, vaccine), and the function below of incidence also makes use of the anticipated time at risk for each participant τ.

$$\varphi(\lambda_T) = \lambda_T{}^2 / (1 - e^{-\lambda_T * \tau}) \qquad (2)$$

With cumulative recurrence of 30% in three years, we implement the formula for cumulative recurrence of $F(\tau) = 1\text{-}e^{-\lambda\tau}$, to determine annual incidence of recurrence in the absence of vaccination to be $\lambda_C = -\ln(1-F(\tau))/\tau = -\ln(1-.30)/3 = 12\%$. With an anticipated hazard ratio of 0.5, then our annual hazard of recurrence in the vaccine arm is λ_I = 0.5*12%= 6%, and the cumulative incidence in the vaccine arm over 3 years is $1\text{-}e^{-.06*3}$, or 16%.

Here is an alternative using cumulative proportions that recur by the end of the follow-up rather than time to event outcomes:

$$2N = 2\left\{Z_\alpha\sqrt{\bar{p}(1-\bar{p})} + Z_\beta\sqrt{p_C(1-p_C) + p_I(1-p_I)}\right\}^2 / (p_C - p_I)^2$$

If we used instead the simple formula above for a difference of two proportions,[18] and used the cumulative proportion recurred after three years ($F(\tau)$ in place of p) then the sample size would come to 354, only about 14% more than the 310 we compute using the survival formula. This comparability assures us that 18-month visit windows (between screening at -1, 18, and 36 months) are sufficient for assessing a difference in time to recurrence detection. When not considering timing of event, a binary-based comparison of cumulative recurrence rates is almost as powerful.

14.2 Randomization

Participants will be randomized 1:1 to receive vaccine or placebo as equal randomization is the most efficient approach to trial design as it minimizes sample size and maximizes information obtained on both arms.[19] See Section 5.8 for details of randomization.

15 STATISTICAL CONSIDERATIONS

15.1 Missing data and loss to follow-up

As mentioned above, we will enroll 35 additional participants so that if 10% of our planned cohort does not complete 3 years of recurrence assessments, we will still be powered to detect our aims of interest. Nonetheless, as described in the protocol, efforts will be made to contact participants who do not appear for scheduled visits, and they will be encouraged to continue their study commitment. Loss to follow-up will be monitored and compared between arms, and reasons for discontinuation will be recorded, if available. We will measure potential differential follow-up by vaccination status, and the potential for loss to follow-up to be associated with the study outcome (either those with recurrence are more likely or less likely to attend). Methods for evaluation of the primary aim may differ depending on whether differential follow-up is observed.

15.2 Visit windows

Participants will undergo surveillance for HSIL at 0, 18 and 36 months, using visualization via anoscopy or vulvoscopy, with a biopsy if a lesion is detected at 0 or 18 months. All participants will receive a scope-directed biopsy at the last screening visit (36 months). As recurrences are anticipated to be detected early by study surveillance, it is unlikely that participants will present for earlier diagnosis between scheduled visits, but information on HSIL diagnosed outside the study will be collected from medical records. In analysis, we will use timing of detection of the recurrence rather than imputing the possible timing of recurrence itself. While the time between visit windows is 18 months, comparison of survival-type and cumulative incidence type sample size calculations demonstrate that obtaining the timing of event is not critical to determining a difference in hazard of event by arm. With annual incidence of recurrence λ_C = 12%, the median time to recurrence is $-\ln(1-.5)/.12 = 5.8$ years. On the vaccine it will take twice as long, 11.7 years. These large differences do not require short visit windows to detect recurrences.

16 STATISTICAL ANALYSIS PLAN

16.1 Data handling

We anticipate some participants will miss visits, which adds uncertainty to the timing of events. For those who have a recurrence detected following a missed visit(s), a sensitivity analysis will be performed setting the timing of the recurrence first to its detection date and secondly to the date of that earliest missed visit prior to detection. Other violations, such as failure to complete all vaccine doses, will be handled by comparison of the Intention to Treat (ITT) and Per Protocol (PP) analyses.

16.2 Analysis populations

The ITT population will be those enrolled and randomized and for whom events accrue starting at day 30 after the first dose of the vaccine. The PP population will be those who completed all phases of vaccine and did not have a subsequent HSIL within 1 month of the last dose. These persons in the PP population will also be required to have an HPV type that is consistent with the vaccine types. Efficacy analyses will be conducted on both the ITT and PP populations, with the ITT population considered primary. The safety population is the same as the ITT population.

16.3 Primary endpoint

We will evaluate differences in the hazard of recurrence using Cox proportional hazards in the ITT population and the PP population, if appropriate; see contingencies for departures from model assumptions below. As this is a randomized trial, the primary analysis will include no other potential predictors of recurrence except for the treatment arm: vaccine *vs.* placebo. However, additional known risk factors will be included in secondary analyses of the primary aim, to confirm findings and to adjust for potential imbalances that occurred during randomization.

In secondary analyses we will adjust for factors upon which participants were stratified: anatomic site, HIV status (positive vs negative), time since diagnosis (<12 months, >12 months). We will also assess balance with regard to smoking status and include it in secondary models. We will assess whether to include smoking as a binary covariate, and we will assess using current consumption levels among smokers. Graphical depictions of endpoint rates by levels of smoking can help determine appropriate parameterization.

16.4 Secondary endpoints

Secondary analyses will include additional exposures or on subsets.

Sub-Aim 1.
For Sub-Aim 1, we will evaluate whether risk of recurrence is more frequent among those with HPV persistence. Persistence will be measured by HPV genotype or HPV16 variant lineage from swab samples collected at months 0, 18 and 36. We will use Cox proportional hazards, but including a time-dependent covariate of HPV persistence by DNA detection from swab samples. Here, we will include vaccination status in the model and include HPV persistence as both a main effect and an interaction term. This will allow us to simultaneously determine whether the presence of HPV DNA impacts risk of recurrence independently of vaccination. We will assess the impact of persistence also among those with different HPV16 lineages, with interaction terms between HPV lineage and vaccination status.

Sub-Aim 2.
We will divide the study data by treatment arm in order to assess Sub-Aim 2, evaluating placebo and vaccine recipients separately. We will assess whether presence and amount of HPV antibody, detected at baseline in the placebo arm, is protective against recurrence. Subsequent graphical analysis examining incidence of recurrence by quartiles and deciles of antibody level will allow visual assessment of the relationship between levels of antibody and risk of recurrence. In further exploratory analyses for this sub-Aim, for the vaccine arm, we will assess whether magnitude of vaccine antibody levels month 1 following the third vaccination in the vaccine arm affects recurrence. Should a cutoff be observed, we may be able to determine the level of antibody (by titer) associated with protection.

Sub-Aim 3.
We will compare the frequency of HSIL recurrences among those vaccinated *vs.* those who received placebo, so that we compare the rate of recurrence in each arm of the study. In contrast to other aims, this analysis will include all recurrences, not just first recurrences. We will do this using Poisson regression with a robust variance estimate, comparing the total number of recurrences for on each arm over the total follow-up per

arm. This is done in part to mitigate the potential influence of early recurrences which occur prior to the full benefit of the vaccine.

16.5 Departures from model assumptions

If the hazards of recurrence by arm are not proportional but do not cross, a log rank test is most powerful to detect the alternative hypothesis of vaccine benefit. However, should the hazards appear to cross, we will make use of current powerful methods, such as those suggested by Liu or Li.[20,21] If informative censoring is suspected, alternative analytical methods may be employed such as a recent one by Zhao which address informative censoring through imputation, without modeling the underlying censoring mechanism[6].

16.6 Left truncation

Some persons will not be eligible for inclusion in per-protocol analysis of the primary aim because their recurrence occurs prior to 1 month post third vaccination. This affects persons in both arms in a placebo-controlled trial. Should the vaccine be partially effective after only one or two doses, there may be a smaller number of persons recurring during the vaccination series in the active vaccine arm versus the placebo arm. Comparison of the included populations and of the results between the ITT and PP analyses will help elucidate this possibility.

17 REFERENCES

1. Saraiya M, Unger ER, Thompson TD, Lynch CF, Hernandez BY, Lyu CW, Steinau M, Watson M, Wilkinson EJ, Hopenhayn C, Copeland G, Cozen W, Peters ES, Huang Y, Saber MS, Altekruse S, Goodman MT. US Assessment of HPV Types in Cancers: Implications for Current and 9-Valent HPV Vaccines. *J Natl Cancer Inst* 2015;107

2. Jones RW, Rowan DM, Stewart AW. Vulvar intraepithelial neoplasia: aspects of the natural history and outcome in 405 women. *Obstet Gynecol* 2005;106:1319-26.

3. Carter JJ, Madeleine MM, Shera K, Schwartz SM, Cushing-Haugen KL, Wipf GC, Porter P, Daling JR, McDougall JK, Galloway DA. Human papillomavirus 16 and 18 L1 serology compared across anogenital cancer sites. *Cancer Res* 2001;61:1934-40.

4. Jones RW. Vulval intraepithelial neoplasia: current perspectives. *Eur J Gynaecol Oncol* 2001;22:393-402.

5. Fehr MK, Baumann M, Mueller M, Fink D, Heinzl S, Imesch P, Dedes K. Disease progression and recurrence in women treated for vulvovaginal intraepithelial neoplasia. *J Gynecol Oncol* 2013;24:236-41. PMCID: PMC3714461

6. Longo WE, Vernava AM, III, Wade TP, Coplin MA, Virgo KS, Johnson FE. Recurrent squamous cell carcinoma of the anal canal. Predictors of initial treatment failure and results of salvage therapy. *Ann Surg* 1994;220:40-9. PMCID: PMC1234285

7. van Seters M., van Beurden M., de Craen AJ. Is the assumed natural history of vulvar intraepithelial neoplasia III based on enough evidence? A systematic review of 3322 published patients. *Gynecol Oncol* 2005;97:645-51.

8. Swedish KA, Factor SH, Goldstone SE. Prevention of recurrent high-grade anal neoplasia with quadrivalent human papillomavirus vaccination of men who have sex with men: a nonconcurrent cohort study. *Clin Infect Dis* 2012;54:891-8.

9. Fleshner PR, Chalasani S, Chang GJ, Levien DH, Hyman NH, Buie WD. Practice parameters for anal squamous neoplasms. *Dis Colon Rectum* 2008;51:2-9.

10. Miltz A, Price H, Shahmanesh M, Copas A, Gilson R. Systematic Review and Meta-Analysis of L1-VLP-Based Human Papillomavirus Vaccine Efficacy against Anogenital Pre-Cancer in Women with Evidence of Prior HPV Exposure. *PLoS One* 2014;9:e90348. PMCID: PMC3940851

11. Kang WD, Choi HS, Kim SM. Is vaccination with quadrivalent HPV vaccine after loop electrosurgical excision procedure effective in preventing recurrence in patients with high-grade cervical intraepithelial neoplasia (CIN2-3)? *Gynecol Oncol* 2013;130:264-8.

12. Joura EA, Giuliano AR, Iversen OE, Bouchard C, Mao C, Mehlsen J, Moreira ED, Jr., Ngan Y, Petersen LK, Lazcano-Ponce E, Pitisuttithum P, Restrepo JA, Stuart G, Woelber L, Yang YC, Cuzick J, Garland SM, Huh W, Kjaer SK, Bautista OM, Chan IS, Chen J, Gesser R, Moeller E, Ritter M, Vuocolo S, Luxembourg A. A 9-Valent HPV Vaccine against Infection and Intraepithelial Neoplasia in Women. *N Engl J Med* 2015;372:711-23.

13. Castellsague X, Giuliano AR, Goldstone S, Guevara A, Mogensen O, Palefsky JM, Group T, Shields C, Liu K, Maansson R, Luxembourg A, Kaplan SS. Immunogenicity and safety of the 9-valent HPV vaccine in men. *Vaccine* 2015;33:6892-901.

14. Van Damme P, Olsson SE, Block S, Castellsague X, Gray GE, Herrera T, Huang LM, Kim DS, Pitisuttithum P, Chen J, Christiano S, Maansson R, Moeller E, Sun X, Vuocolo S, Luxembourg A. Immunogenicity and Safety of a 9-Valent HPV Vaccine. *Pediatrics* 2015;136:e28-e39.

15. Kahn JA, Xu J, Kapogiannis BG, Rudy B, Gonin R, Liu N, Wilson CM, Worrell C, Squires KE. Immunogenicity and safety of the human papillomavirus 6, 11, 16, 18 vaccine in HIV-infected young women. *Clin Infect Dis* 2013;57:735-44. PMCID: PMC3739463

16. Slade BA, Leidel L, Vellozzi C, Woo EJ, Hua W, Sutherland A, Izurieta HS, Ball R, Miller N, Braun MM, Markowitz LE, Iskander J. Postlicensure safety surveillance for quadrivalent human papillomavirus recombinant vaccine. *JAMA* 2009;302:750-7.

17. Lachin JM. Introduction to sample size determination and power analysis for clinical trials. *Control Clin Trials* 1981;2:93-113.

18. Friedman LM, Furberg CD, DeMets DL. Fundamentals of clinical trials. 4th New York: Springer (New York); 2010.

19. Lachin JM. Properties of simple randomization in clinical trials. *Control Clin Trials* 1988;9:312-26.

20. Liu K, Qiu P, Sheng J. Comparing two crossing hazard rates by Cox proportional hazards modelling. *Stat Med* 2007;26:375-91.

21. Li H, Han D, Hou Y, Chen H, Chen Z. Statistical inference methods for two crossing survival curves: a comparison of methods. *PLoS One* 2015;10:e0116774. PMCID: PMC4304842

APPENDIX A: STUDY VISIT WINDOWS

VIVA Visit Windows
***Month defined as 30 days*

Visit	STUDY MONTH	VIST WINDOW
0	Screening	--
1	Enrollment/Month 0 (Dose 1)	Within 56 days of screen
2	Month 2 Visit (Dose 2)[1]	at least 28 days from Dose 1
3	Month 6 Visit (Dose 3)[2]	at least 3 months from Dose 2<br>AND<br>At least 5 months from Dose 1
4	Month 7 Visit[3]	+14 days
5	Month 12 Visit (phone)	- 60 / + 90 days
6	Month 18 Visit	- 60 / + 90 days
7	Month 24 Visit (phone)	- 60 / + 90 days
8	Month 36 Visit	- 60 / + 90 days
9	Month 42 Visit (phone)	- 60 / + 90 days

[1] Dose 2 can be given at a maximum allowable time of 9 months post Dose 1
[2] Dose 3 can be given at a maximum allowable time of 12 months post Dose 1.
[3] The Month 7 Visit is targeted for 1 month after Dose 3.

References:
https://www.cdc.gov/mmwr/preview/mmwrhtml/rr6305a1.htm
http://www.immunize.org/askexperts/experts_hpv.asp

The minimum interval between the first and second doses of vaccine is 4 weeks. The minimum interval between the second and third doses of vaccine is 12 weeks. The minimum interval between the first and third doses is 5 calendar months. If the vaccination series is interrupted, the series does not need to be restarted.

APPENDIX B: TABLE OF PROCEDURES

			Study Month							
	**Screen (-1)**	**Enrollment (Month 0)**	**2**	**6**	**7[1]**	**12**	**18**	**24**	**36**	**42**
Obtain Informed Consent	X									
Review Inclusion/Exclusion Criteria	X	X								
Medical History and PE	X									
Interim History and Targeted PE		X	X	X	X		X		X	
HIV Testing (as needed)	X									
Urine Pregnancy Test (women only)	X	X	X	X						
Randomization		X								
Vaccination		**X**	**X**	**X**						
Self-Administered Questionnaire		X		X			X		X	
Blood Draw - Serum (up to 20 cc)		X			X		X		X	
Blood Draw for DNA (optional)					X					
Oral Rinse	X				X		X		X	
Anoscopy/Vulvoscopy	X[2]						X		X	
Swab for HPV	X						X		X	
Biopsy	X[3]						X[3]		X[4]	
Telephone Visit						X		X		X

[1] Reminder calls will be conducted every 1-3 months after the Month 7 visit for study retention.
[2] Anoscopy/vulvoscpy will not be performed if the procedure was done within 8 weeks of the Screening Visit with either Dr. Constance Mao, Dr Jeffrey Schouten, or an ANCHOR-certified provider.
[3] Biopsy, as needed, if lesion is noted at qualifying site during anoscopy/vulvoscopy
[4] A biopsy will be performed at site of any visible lesion or at the site of qualifying lesion if no lesion is visible.
